# Study on Modelling Method of Resilient Mat Used under Floating Slab Track

**DOI:** 10.3390/ma16083078

**Published:** 2023-04-13

**Authors:** Zhuosheng Xu, Xiaojing Sun, Chang Qiao, Tingting Wang, Meng Ma

**Affiliations:** 1 Key Laboratory of Urban Underground Engineering of Ministry of Education, Beijing Jiaotong University, Beijing 100044, China; 2 School of Civil Engineering, Beijing Jiaotong University, Beijing 100044, China; 3 Beijing Jiuzhouyigui Environmental Technology Co., Ltd., Beijing 100071, China

**Keywords:** railway vibration, resilient mat, finite element, floating slab track, 3PVM

## Abstract

Kelvin’s model is widely used to simulate the dynamic characteristic of a resilient mat under a slab track. To develop an effective calculation model for a resilient mat using a solid element, a three-parameter viscoelasticity model (3PVM) was employed. With the help of the user-defined material mechanical behavior, the proposed model was implemented in software ABAQUS. To validate the model, a laboratory test was performed on a slab track with a resilient mat. Then, a finite element model of the track-tunnel-soil system was built. The calculation results using the 3PVM was compared with those using Kelvin’s model and the test results. The results indicate that the 3PVM can better reflect the dynamic characteristics of resilient mat than Kelvin’s model, especially over 10 Hz. Compared with the test results, the 3PVM has an average error of 2.7 dB and a max error of 7.9 dB at 5 Hz.

## 1. Introduction

With the development of urban rail transit, the problems of train-induced tunnel and building vibrations have received increasing attention recently [1,2,3,4,5]. These environmental issues have potential impacts on the nearby residents [6,7,8,9], the long-term protection of historic buildings [10,11,12,13], and the operation of precision instruments [14,15]. To control the train-induced vibrations, various types of mitigation measures can be taken: wheel profiling and rail grinding [16,17,18,19], designing vibration mitigation measures on the track [20,21], using a propagation path [22,23], and using vibration receivers [24].

As a type of track solution, the floating slab track (FST) has been proven to be the most effective. Springs or resilient mats can be installed under the slab, forming a mass-spring system. A large number of scholars have carried out theoretical and experimental research on a steel spring FST [8,25,26,27,28]. The FST with a slab mat has also gained attention. Auersch et al. (2012) [29] analyzed the reduction in ground vibration by the FST with a slab mat. Jin et al. (2015) [30] performed an experimental study to compare the vibration reduction ability of the rubber FSTs with different supporting forms. Jin et al. (2017) [31] also optimized the vibration reduction in the rubber FSTs using the multi-island genetic algorithm. He et al. (2021) [32] performed field measurements on the noise and vibrations of the elevated urban rail transit line where the FST with rubber mats was installed.

In the analytical and numerical FST model, steel springs can be reasonably simulated by Kelvin’s element. Although the resilient slab mats are far different from the steel spring due to its special viscoelasticity, in practice they are still widely modelled by spring elements or Kelvin’s elements. Lombaert et al. (2006) [33] analyzed the effectiveness of an FST for the control of ground-borne vibrations. In this model, the slab mat was assumed to act as a set of distributed, independent linear springs and dampers, with vertical stiffness. Xin and Gao (2011) [34] and Xin et al. (2020) [35] established a train-track coupling model, in which the slab mats were modelled by Kelvin’s elements. Qu et al. (2019) [36] analyzed the vibration mitigation characteristics of the ballasted ladder track with elastic elements. Both of the under sleeper pads (USPs) and under ballast mats (UBMs) were simulated by elastic spring elements. Liang et al. (2020) [37] proposed a frequency-domain theoretical model of a vehicle-track coupling interaction system, in which the resilient mats were simplified as vertical support springs. Hou et al. (2022) [38] evaluated the vibration reduction effects of ballast mats by numerical and experimental analysis. In the wheel-track model, the ballast mats were modelled by Kelvin’s elements.

The Kelvin’s element is composed of a linear spring and a viscous damper in parallel. It is a simple and standard mechanical model. However, the resilient mat is usually made of viscoelastic polymer material, and it is noticed that Kelvin’s model cannot simulate its vibration characteristics well [39]. To solve this problem, the fractional derivative model was introduced, by which the influence of loading history and the frequency variation characteristics of resilient mats can be considered. Accordingly, it was introduced to model the rail pads and slab mats in vehicle-track models [40,41,42,43,44].

The Maxwell element is composed of a linear spring and a viscous damping in series. The frequency increase can cause a linear decrease in the loss factor of the Maxwell element. In addition, the Maxwell element cannot reflect the creep characteristic of solid material, which means it is not suitable to simulate the resilient mat. The burgers element is composed of the Maxwell element and Kelvin’s element in series. Thus, the Burgers element also cannot simulate solid material well.

It can be found that the simulations of resilient mats in the above research were based on the combination of a linear spring and a viscous damping in different ways. However, in finite element (FE) software(ABAQUS CAE), when the geometric form of the resilient mat needs to be considered, the solid element is often the most convenient and simplest. Obviously, the solid element based on the traditional elastic constitutive model cannot describe the dynamic characteristics of the slab mats well. Then, a three-parameter viscoelastic model (3PVM) is employed in the present study, which is used to simulate the slab mats with solid elements in the FE software(ABAQUS CAE). To verify the accuracy of the proposed model, a dynamic test was performed on the FST with resilient pads in the laboratory. The test results were used to compare the difference between the proposed model and the other two traditional modeling methods.

## 2. The 3PVM and Implemented in FE Software

### 2.1. Constitutive Relation for 3PVM

**Figure 1:**
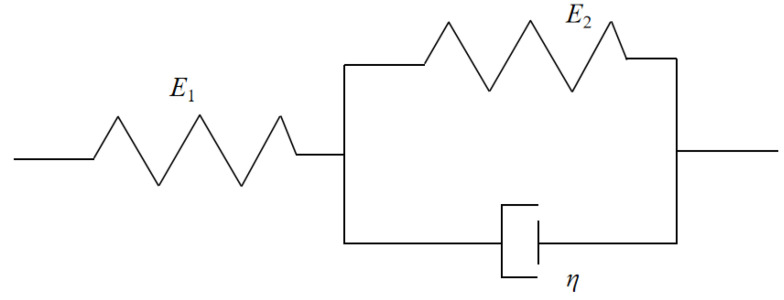
The relationship between subscription 1 and 2.

A basic 3PVM can be obtained by a Kelvin’s model and a spring in series. It can characterize the mechanical properties of viscoelastic materials well. The constitutive equations can be expressed as

(1) $$\begin{array}{l} \varepsilon =\varepsilon_{1}+\varepsilon_{2}\\ \sigma =E_{1}\varepsilon_{1}+\eta {\dot{\varepsilon }}_{1}\\ \sigma =E_{2}\varepsilon_{2} \end{array}$$

where *σ* and *ε* are stress and strain; *η* is viscosity coefficient; *E* is modulus of elasticity; and $\dot{\varepsilon }=d\varepsilon /dt$ is strain rate; the relationship between subscripts 1 and 2 is described in Figure 1.

Based on Equation (1), the following equations can be obtained:

(2) $$\sigma +\frac{\eta }{E_{1}+E_{2}}\dot{\sigma }=\frac{1}{1/E_{1}+1/E_{2}}\varepsilon +\frac{\eta }{1+E_{1}/E_{2}}\dot{\varepsilon }$$

By the Fourier transform of Equation (2), the complex modulus and loss factor of the material can be obtained in the frequency domain:

(3) $$\begin{array}{l} E^{\prime }(\omega )=\frac{E_{1}{}^{2}E_{2}+E_{1}E_{2}{}^{2}+E_{2}\eta^{2}\omega^{2}}{{\left(E_{1}+E_{2}\right)}^{2}+\eta^{2}\omega^{2}}\\ E^{\prime \prime }(\omega )=\frac{E_{2}{}^{2}\eta \omega }{{\left(E_{1}+E_{2}\right)}^{2}+\eta^{2}\omega^{2}}\\ \tan\delta =\frac{E_{2}\eta \omega }{E_{1}{}^{2}+E_{1}E_{2}+\eta^{2}\omega^{2}} \end{array}$$

It can be observed that the dynamic modulus and loss factor of the three parameters are functions of the frequency *ω*. Therefore, this model can represent the frequency-dependent characteristics of viscoelastic materials. When the parameters of viscoelastic materials at different frequencies are known, the nonlinear least square method can be used to fit the above equation, and the material parameters in the constitutive equation can be obtained. The slab mat is a type of solid viscoelastic material, with typical frequency-dependent characteristics of the dynamic mechanical parameters, i.e., the complex modulus and the loss factor. Accordingly, this model is suitable for modelling resilient mats.

To better represent the loading history of viscoelastic materials, the integral constitutive equations are usually used. Generally, the loading process of materials is complex, but linear materials can be represented by the linear superposition of simple loads. When the load Δ*σ*_1_ is applied at time *t*_1_, the strain is substituted into the creep equation as follows:

(4) $$\varepsilon (t)=J(t)\sigma_{0}+\Delta \sigma_{1}J(t-t_{1})$$

where *J*(*t*) is the creep function and $\sigma_{0}$ is constant stress. Assuming that there are *r* stress increments acting on the material alone at this time, the time strain after this time can be expressed as

(5) $$\varepsilon (t)=J(t)\sigma_{0}+\sum_{i=1}^{r}\Delta \sigma_{i}J(t-t_{1})$$

Based on the above principles, assuming that the stress load function is continuously differentiable, it can be decomposed into the action of the sum of one stress *σ*_0_*H*(*t*) and countless small stresses *dσ*(*ξ*)*H*(*t* − *ξ*) through the differential principle. Then, the strain at time t can be expressed as

(6) $$\varepsilon (t)=\sigma_{0}J(t)+\int_{0}^{t}J(t-\xi )\frac{d\sigma (\xi )}{d\xi }d\xi$$

If a sudden stress is applied at time *t*_1_, the strain value generated by this stress is

(7) $$\Delta \varepsilon (t)=J(t-t_{1})\Delta \sigma H(t-t_{1})$$

Then, the creep integral constitutive equations can be obtained:

(8) $$\varepsilon (t)=\int_{-\infty }^{t}J(t-\xi )\frac{d\sigma (\xi )}{d\xi }d\xi$$

Similarly, the constitutive equation in the form of stress relaxation can be written as

(9) $$\sigma (t)=\int_{-\infty }^{t}Y(t-\xi )\frac{d\sigma (\xi )}{d\xi }d\xi$$

where *Y*(*t*) is relaxation function or relaxation modulus.

It can be observed that the integral constitutive relation can clearly reflect the loading history of viscoelastic materials. Extending the constitutive equation to the three-dimensional (3D) case: assuming that the material is isotropic, the viscoelastic constitutive equation in the 3D state can be expressed as

(10) $$\begin{array}{c} s_{ij}(t)=\int_{0}^{t}2G(t-\xi )\frac{\partial e_{ij}(\xi )}{\partial \xi }d\xi \\ \sigma_{kk}(t)=\int_{0}^{t}3K(t-\xi )\frac{\partial \varepsilon_{kk}(\xi )}{\partial \xi }d\xi \end{array}$$

or

(11) $$\begin{array}{ll} \sigma_{ij}(t) & =s_{ij}(t)+\frac{\delta_{i}{}_{j}}{3}\sigma_{kk}(t)\\ & =\int_{0}^{t}2G(t-\xi )\frac{\partial e_{ij}}{\partial \xi }d\xi +\frac{\delta_{i}{}_{j}}{3}\int_{0}^{t}3K(t-\xi )\frac{\partial \varepsilon_{kk}}{\partial \xi }d\xi \end{array}$$

where *G*(*t*) and *K*(*t*) is shear relaxation function and bulk relaxation function. *G*(*t)* and *K*(*t)* can be defined by *Y*(*t)*:

(12) $$\begin{array}{l} G(t)=\frac{Y(t)}{2(1+\gamma )}\\ K(t)=\frac{Y(t)}{3(1-2\gamma )} \end{array}$$

### 2.2. Implementation of 3PVM in ABAQUS

The FE software ABAQUS was used to analyze the dynamic responses of the FST. To model the resilient mat using the 3PVM, the material properties need to be customized in ABAQUS. Based on the integral constitutive equation of the 3PVM proposed in Section 2.1, it is necessary to derive the uniform tangent stiffness matrix (Jacobian matrix) of the material. Then, combined with the implementation technology of UMAT subroutine interface and user-defined material mechanical behavior in ABAQUS, FORTRAN statements are used to write relevant programs for ABAQUS main program to call.

**Figure 2:**
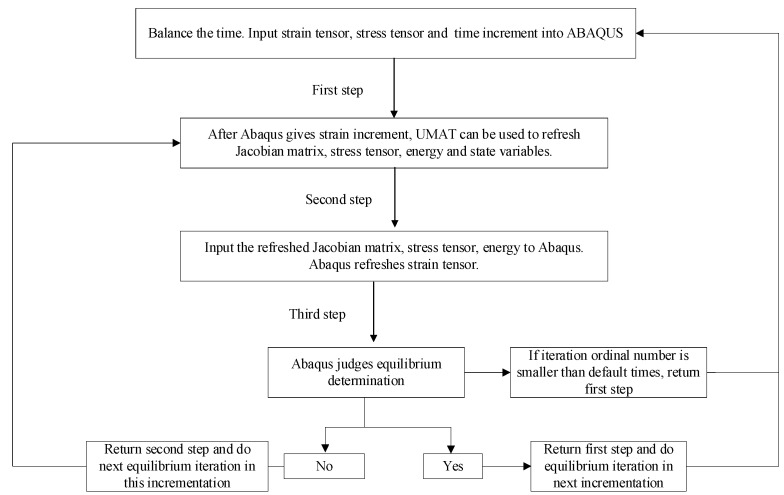
ABAQUS calls UMAT subroutine.

The key of compiling the UMAT subroutine is that the user provides the Jacobian matrix of the material constitutive model and updates the information of stress tensor, energy, etc., to the ABAQUS main program. The ABAQUS main program calls UMAT subroutine and cooperates with it, as illustrated in Figure 2.

If the 3PVM model is used to simulate the solid resilient mats, the 3D constitutive equation must be used. Firstly, the 3PVM constitutive equation can be simplified as

(13) $$\sigma +p_{1}\dot{\sigma }=q_{0}+q_{1}\dot{\varepsilon }$$

where *p*_1_ = *η*/(*E*_1_ + *E*_2_), *q*_0_ = 1/(1/*E*_1_ + 1/*E*_2_), and *q*_1_ = *η*/(1 + *E*_1_/*E*_2_). Then, the relaxation function of the 3PVM can be written as

(14) $$Y(t)=q_{0}+(\frac{q_{1}}{p_{1}}-q_{0})e^{-t/p_{1}}$$

In the 3D state, the relationship among shear relaxation modulus *G*(*t*), relaxation modulus *Y*(*t*), and bulk relaxation modulus *K*(*t*) are expressed as

(15) $$\begin{array}{l} G(t)=\frac{Y(t)}{2(1+\nu )}\\ K(t)=\frac{Y(t)}{3(1-2\nu )} \end{array}$$

Substitute Equation (14) into Equation (15),

(16) $$\begin{array}{l} G(t)=\frac{1}{2(1+\nu )}[q_{0}+(\frac{q_{1}}{p_{1}}-q_{0})e^{-t/p_{1}}]\\ K(t)=\frac{1}{3(1-2\nu )}[q_{0}+(\frac{q_{1}}{p_{1}}-q_{0})e^{-t/p_{1}}] \end{array}$$

Then, by substituting Equation (16) into Equation (10), the deviatoric and sphere tensor of the 3PVM can be obtained:

(17) $$\begin{array}{ll} s_{ij}(t) & =\frac{1}{1+\nu }\int_{0}^{t}\left[q_{0}+(\frac{q_{1}}{p_{1}}-q_{0})e^{-(t-\xi )/p_{1}}\right]\frac{\partial e_{ij}(\xi )}{\partial \xi }d\xi \\ & =\frac{q_{0}}{1+\nu }e_{ij}(t)+\frac{1}{1+\nu }(\frac{q_{1}}{p_{1}}-q_{0})\int_{0}^{t}e^{-(t-\xi )/p_{1}}\frac{\partial e_{ij}(\xi )}{\partial \xi }d\xi \end{array}$$

(18) $$\begin{array}{ll} \sigma_{kk}(t) & =\frac{1}{1-2\nu }\int_{0}^{t}\left[q_{0}+(\frac{q_{1}}{p_{1}}-q_{0})e^{-(t-\xi )/p_{1}}\right]\frac{\partial \varepsilon_{kk}(\xi )}{\partial \xi }d\xi \\ & =\frac{q_{0}}{1-2\nu }\varepsilon_{kk}(t)+\frac{1}{1-2\nu }(\frac{q_{1}}{p_{1}}-q_{0})\int_{0}^{t}e^{-(t-\xi )/p_{1}}\frac{\partial \varepsilon_{kk}(\xi )}{\partial \xi }d\xi \end{array}$$

The above two equations are the sum of a constant term and an integral term. For convenience, *s_ij_*_,0_(*t*), *s_ij_*_,1_(*t*), *σ_kk_*_,0_(*t*), and *σ_kk_*_,1_(*t*) are, respectively, used to represent the constant term and integral term of stress partial tensor and spherical tensor, namely,

(19) $$s_{ij,0}(t)=\frac{q_{0}}{1+\nu }e_{ij}(t)$$

(20) $$s_{ij,1}(t)=\frac{1}{1+\nu }(\frac{q_{1}}{p_{1}}-q_{0})\int_{0}^{t}e^{-(t-\xi )/p_{1}}\frac{\partial e_{ij}(\xi )}{\partial \xi }d\xi$$

(21) $$\sigma_{kk,0}(t)=\frac{q_{0}}{1-2\nu }\varepsilon_{kk}(t)$$

(22) $$\sigma_{kk,1}(t)=\frac{1}{1-2\nu }(\frac{q_{1}}{p_{1}}-q_{0})\int_{0}^{t}e^{-(t-\xi )/p_{1}}\frac{\partial \varepsilon_{kk}(\xi )}{\partial \xi }d\xi$$

At time (*t* + Δ*t*), the partial stress tensor and spherical tensor are

(23) $$s_{ij,0}(t+\Delta t)=\frac{q_{0}}{1+\nu }e_{ij}(t+\Delta t)$$

(24) $$s_{ij,1}(t+\Delta t)=\frac{1}{1+\nu }(\frac{q_{1}}{p_{1}}-q_{0})\int_{0}^{t+\Delta t}e^{-(t+\Delta t-\xi )/p_{1}}\frac{\partial e_{ij}(\xi )}{\partial \xi }d\xi$$

(25) $$\sigma_{kk,0}(t+\Delta t)=\frac{q_{0}}{1-2\nu }\varepsilon_{kk}(t+\Delta t)$$

(26) $$\sigma_{kk,1}(t+\Delta t)=\frac{1}{1-2\nu }(\frac{q_{1}}{p_{1}}-q_{0})\int_{0}^{t+\Delta t}e^{-(t+\Delta t-\xi )/p_{1}}\frac{\partial \varepsilon_{kk}(\xi )}{\partial \xi }d\xi$$

Equation (24) can be divided into two integrals and expressed as

(27) $$\begin{array}{c} s_{ij,1}(t+\Delta t)=\frac{1}{1+\nu }(\frac{q_{1}}{p_{1}}-q_{0})[\int_{0}^{t}e^{-(t+\Delta t-\xi )/p_{1}}\frac{\partial e_{ij}(\xi )}{\partial \xi }d\xi \\ \;\;\;\;\;\;\;\;\;\;\;\;\;\;\;\;\;\;\;\;\;+\int_{t}^{t+\Delta t}e^{-(t+\Delta t-\xi )/p_{1}}\frac{\partial e_{ij}(\xi )}{\partial \xi }d\xi ] \end{array}$$

The first item on the right of the equal sign of Equation (27) can be calculated as

(28) $$\begin{array}{c} \frac{1}{1+\nu }(\frac{q_{1}}{p_{1}}-q_{0})\int_{0}^{t}e^{-(t+\Delta t-\xi )/p_{1}}\frac{\partial e_{ij}(\xi )}{\partial \xi }d\xi \\ =\frac{1}{1+\nu }(\frac{q_{1}}{p_{1}}-q_{0})e^{-\Delta t/p_{1}}\int_{0}^{t}e^{-(t-\xi )/p_{1}}\frac{\partial e_{ij}(\xi )}{\partial \xi }d\xi \\ =e^{-\Delta t/p_{1}}s_{ij,1}(t) \end{array}$$

When Δ*t* is very small, the strain changes approximately linearly in the time period *t*→Δ*t*. Then, the second term on the right of Equation (27) can be approximately expressed as

(29) $$\begin{array}{l} \frac{1}{1+\nu }(\frac{q_{1}}{p_{1}}-q_{0})\int_{t}^{t+\Delta t}e^{-(t+\Delta t-\xi )/p_{1}}\frac{\partial e_{ij}(\xi )}{\partial \xi }d\xi \\ =\frac{1}{1+\nu }(\frac{q_{1}}{p_{1}}-q_{0})\frac{e_{ij}(t+\Delta t)-e_{ij}(t)}{\Delta t}\int_{t}^{t+\Delta t}e^{-(t+\Delta t-\xi )/p_{1}}d\xi \\ =\frac{1}{1+\nu }(\frac{q_{1}}{p_{1}}-q_{0})\frac{\Delta e_{ij}(t+\Delta t)}{\Delta t}(1-p_{1}e^{-\Delta t)/p_{1}}) \end{array}$$

Then,

(30) $$s_{ij,1}(t+\Delta t)=e^{-\Delta t/p_{1}}s_{ij,1}(t)+\frac{1}{1+\nu }(\frac{q_{1}}{p_{1}}-q_{0})\frac{\Delta e_{ij}(t+\Delta t)}{\Delta t}(1-p_{1}e^{-\Delta t/p_{1}})$$

Similarly, Equation (22) can be further expressed as:

(31) $$\sigma_{kk,1}(t+\Delta t)=e^{-\Delta t/p_{1}}\sigma_{kk,1}(t)+\frac{1}{1-2\nu }(\frac{q_{1}}{p_{1}}-q_{0})\frac{\Delta \varepsilon_{kk}(t+\Delta t)}{\Delta t}(1-p_{1}e^{-\Delta t/p_{1}})$$

Now, all items at time (*t* + Δ*t*) have been obtained, so the stress deviation and volume stress are, respectively, expressed as

(32) $$\begin{array}{l} \;\;\;\;\;\;\;\;\;\;s_{ij}(t+\Delta t)=\frac{q_{0}}{1+\nu }e_{ij}(t+\Delta t)+e^{-\Delta t/p_{1}}s_{ij,1}(t)\\ \;\;\;\;\;\;\;\;\;\;\;\;\;\;\;+\frac{1}{1+\nu }(\frac{q_{1}}{p_{1}}-q_{0})\frac{\Delta e_{ij}(t+\Delta t)}{\Delta t}(1-p_{1}e^{-\Delta t/p_{1}}) \end{array}$$

(33) $$\begin{array}{l} \;\;\;\;\;\;\;\;\;\;\;\sigma_{kk}(t+\Delta t)=\frac{q_{0}}{1-2\nu }\varepsilon_{kk}(t+\Delta t)+e^{-\Delta t/p_{1}}\sigma_{kk,1}(t)\\ \;\;\;\;\;\;\;\;\;\;\;\;\;\;\;\;+\frac{1}{1-2\nu }(\frac{q_{1}}{p_{1}}-q_{0})\frac{\Delta \varepsilon_{kk}(t+\Delta t)}{\Delta t}(1-p_{1}e^{-\Delta t/p_{1}}) \end{array}$$

The increment of stress deviation and volume stress are, respectively, expressed as

(34) $$\begin{array}{l} \Delta s_{ij}(t+\Delta t)=s_{ij}(t+\Delta t)-s_{ij}(t)\\ \;\;\;\;\;\;\;\;\;\;\;\;\;\;\;\;\;\;\;\;\;\;=\frac{q_{0}}{1+\nu }\Delta e_{ij}(t+\Delta t)+(e^{-\Delta t/p_{1}}-1)s_{ij,1}(t)\\ \;\;\;\;\;\;\;\;\;\;\;\;\;\;\;\;\;\;\;\;\;\;+\frac{1}{1+\nu }(\frac{q_{1}}{p_{1}}-q_{0})\frac{\Delta e_{ij}(t+\Delta t)}{\Delta t}(1-p_{1}e^{-\Delta t/p_{1}}) \end{array}$$

(35) $$\begin{array}{l} \Delta \sigma_{kk}(t+\Delta t)=\sigma_{kk}(t+\Delta t)-\sigma_{kk}(t)\\ \;\;\;\;\;\;\;\;\;\;\;\;\;\;\;\;\;\;\;\;\;\;\;=\frac{q_{0}}{1-2\nu }\Delta \sigma_{kk}(t+\Delta t)+(e^{-\Delta t/p_{1}}-1)\sigma_{kk,1}(t)\\ \;\;\;\;\;\;\;\;\;\;\;\;\;\;\;\;\;\;\;\;\;\;\;+\frac{1}{1-2\nu }(\frac{q_{1}}{p_{1}}-q_{0})\frac{\Delta \varepsilon_{kk}(t+\Delta t)}{\Delta t}(1-p_{1}e^{-\Delta t/p_{1}}) \end{array}$$

Accordingly, the stress increment can be obtained

(36) $$\begin{array}{l} \Delta \sigma (t+\Delta t)=\Delta s_{i}{}_{j}(t+\Delta t)+\frac{\delta_{i}{}_{j}}{3}\Delta \sigma_{kk}(t+\Delta t)\\ \;\;\;\;\;\;\;\;\;\;\;\;\;\;\;\;\;\;\;\;=\Delta e_{ij}(t+\Delta t)[\frac{q_{0}}{1+\nu }+\frac{1}{\Delta t(1+\nu )}(\frac{q_{1}}{p_{1}}-q_{0})(1-p_{1}e^{-\Delta t/p_{1}})]\\ \;\;\;\;\;\;\;\;\;\;\;\;\;\;\;\;\;\;\;\;+\frac{\delta_{i}{}_{j}}{3}\Delta \sigma_{kk}(t+\Delta t)[\frac{q_{0}}{1-2\nu }+\frac{1}{\Delta t(1-2\nu )}(\frac{q_{1}}{p_{1}}-q_{0})(1-p_{1}e^{-\Delta t/p_{1}})]\\ \;\;\;\;\;\;\;\;\;\;\;\;\;\;\;\;\;\;\;\;+(e^{-\Delta t/p_{1}}-1)\sigma_{ij,1}(t) \end{array}$$

Rewrite Equation (36) to an expression containing *G*(*t*) and *K*(*t*):

(37) $$\begin{array}{l} \Delta \sigma (t+\Delta t)=(1-\frac{p_{1}e^{-\Delta t/p_{1}}}{\Delta t})[2G(t)\cdot \Delta e_{ij}(t+\Delta t)+3K(t)\cdot \frac{\delta_{i}{}_{j}}{3}\Delta \varepsilon_{kk}(t+\Delta t)]\\ \;\;\;\;\;\;\;\;\;\;\;\;\;\;\;\;\;\;\;\;=(1-\frac{p_{1}e^{-\Delta t/p_{1}}}{\Delta t})D_{ijkl}(t+\Delta t)\varepsilon_{kl}(t+\Delta t)+(e^{-\Delta t/p_{1}}-1)\sigma_{ij}(t) \end{array}$$

Finally, the Jacobian matrix of the material can be obtained

(38) $$DDSDDE_{3\mathrm{PVM}}=(1-\frac{p_{1}e^{-\Delta t/p_{1}}}{\Delta t})\left[\begin{array}{cccccc} \lambda +2\mu & \lambda & \lambda & & & \\ \lambda & \lambda +2\mu & \lambda & & & \\ \lambda & \lambda & \lambda +2\mu & & & \\ & & & \mu & & \\ & & & & \mu & \\ & & & & & \mu \end{array}\right]$$

where *λ* and *μ* are Lame constants.

After obtaining the Jacobian matrix of the material, the FORTRAN compiler can write relevant commands according to the writing format of the UMAT subroutine for the ABAQUS main program to call.

## 3. Dynamic Test on a FST with Resilient Mats

**Figure 3:**
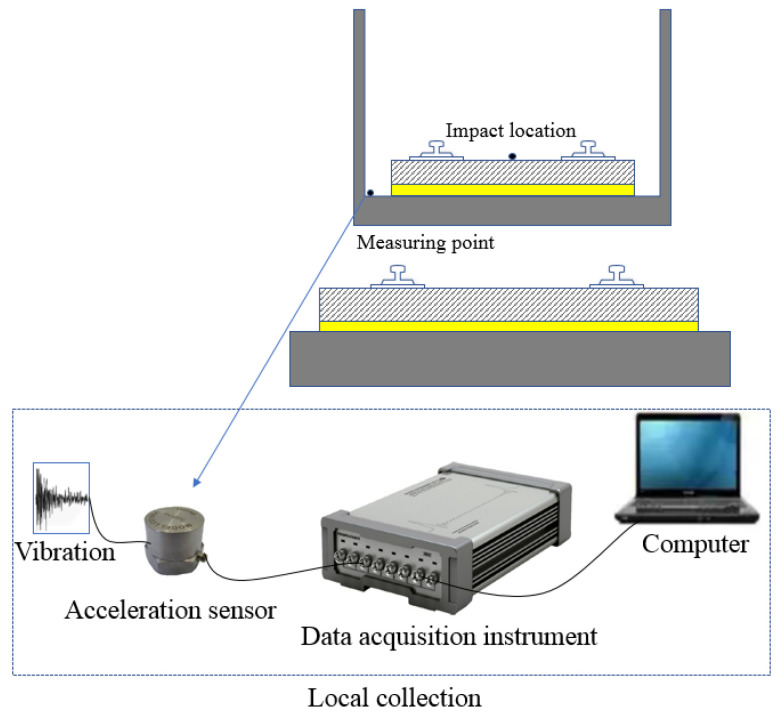
Sketch of laboratory test.

The test was performed in an underground laboratory, where a 7 m × 3.5 m × 0.4 m FST with resilient mats was constructed. To analyze the dynamic behavior of the track system, an automatic falling weight equipment was used to apply the impact load on the center of the track slab. An accelerometer with a range of 5 g and a sensitivity of 987.5 mV/g was installed on the tunnel base (Figure 3).

**Figure 4:**
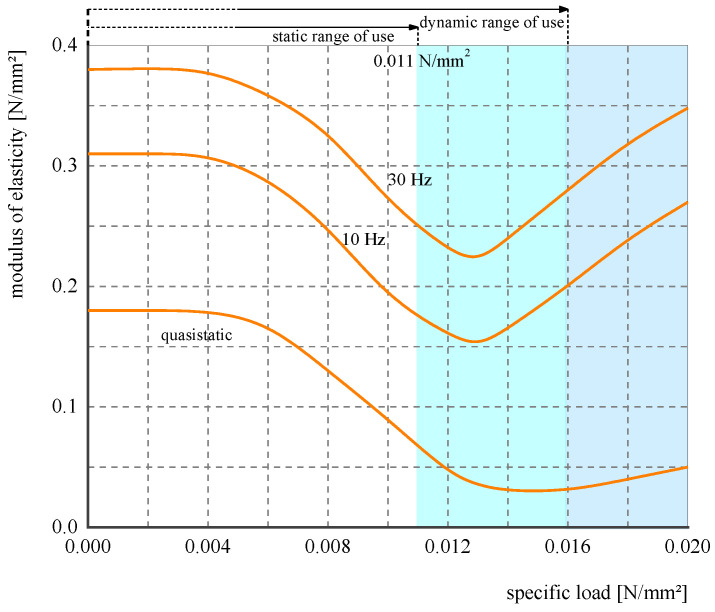
Material characteristic curves.

The area of the resilient mat was 7 m × 3.5 m, in full contact of the slab. Figure 4 illustrates the relationship between the modulus of elasticity and specific load of the test resilient mat, which is provided by the mat manufacturer. Based on the slab dimension of 7 m × 3.5 m × 0.4 m, the total mass of the slab is 25 kg. The total masses of all fasteners and two rails are 2 and 849 kg, respectively. Then, the total weight applying on the mat can be calculated as 0.0104 N/mm^2^.

**Figure 5:**
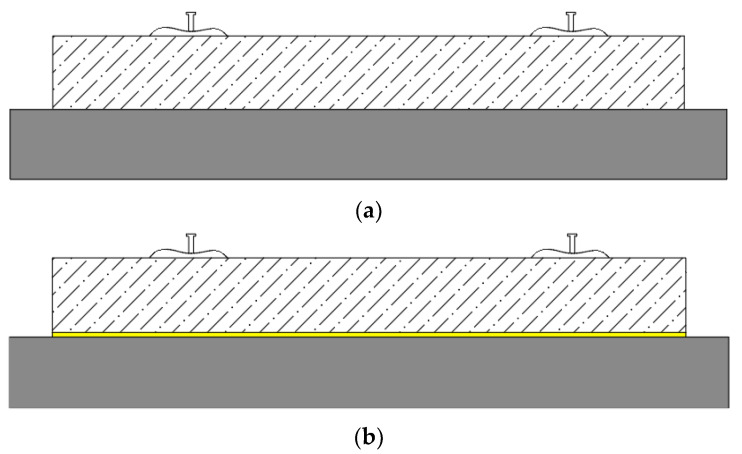
Working condition. (**a**) Without mat. (**b**) With mat.

To analyze vibration mitigation effect and dynamic characteristic of the FST with resilient mats, two test conditions were designed: a slab track without a mat and the FST with a 25 mm-thick fully supported mat, see Figure 5.

The excitation device is a drop-weight hammer, and the impact load applied by hammer can be controlled by the weight of the hammerhead and the height of where the hammerhead is lifted. To control the precision of the impact load, over 200 tests were performed before the formal test, and the bad values are discarded in advance. The transducer and data acquisition equipment are listed in Table 1.

**Figure 6:**
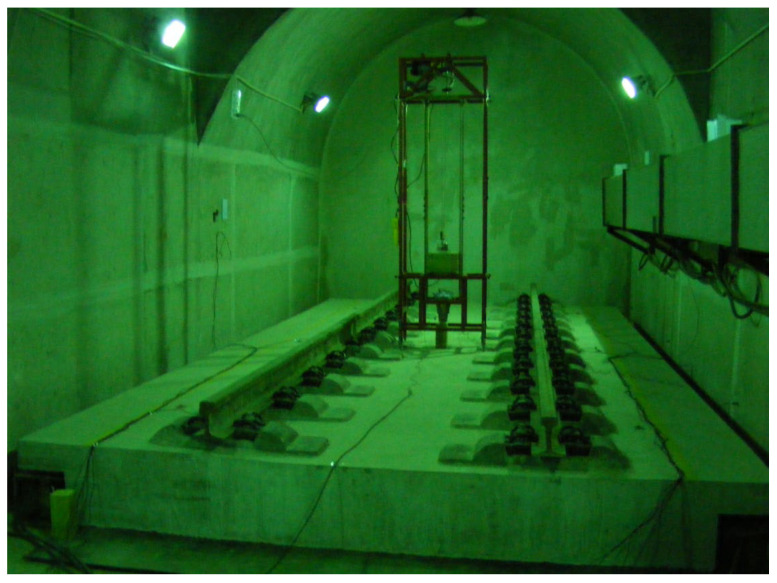
Test site diagram.

**Figure 7:**
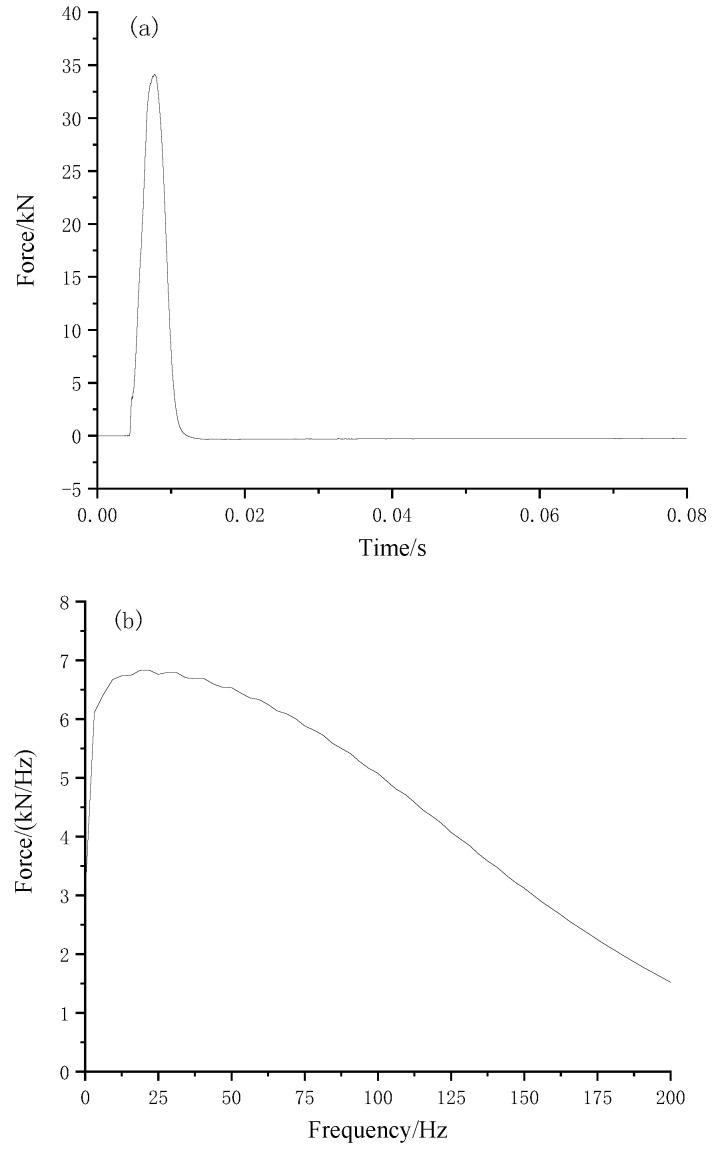
(**a**) Time history and (**b**) Fourier spectrum of the impact load.

The drop-weight hammer was installed on the center of the slab (Figure 6) to generate the vertical impact. The drop weight was 715.4 kN, and the lifting height was 10 cm. The sampling frequency was set as 12.8 kHz. The test was repeated ten times. Figure 7 illustrates the typical time history and Fourier spectrum of the impact load.

**Figure 8:**
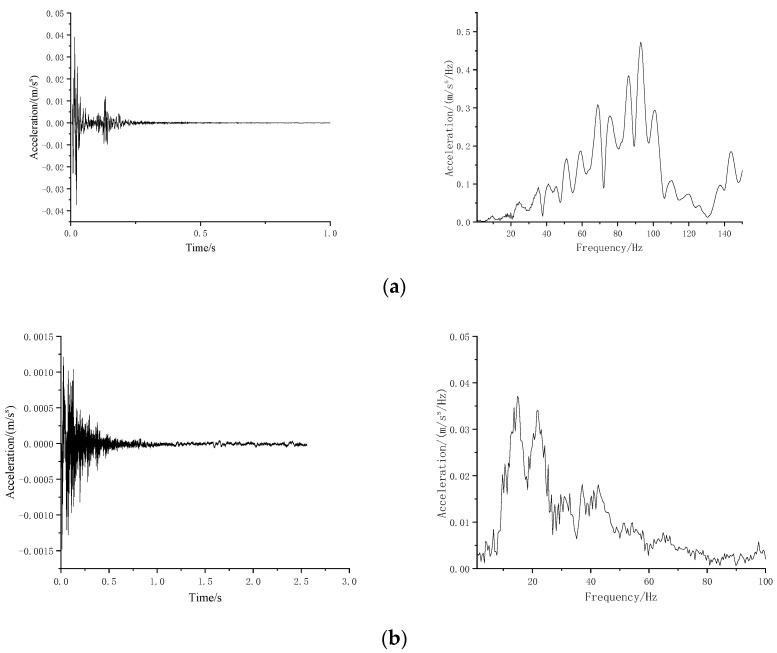
Typical time histories and Fourier spectra of vibration responses for the conditions (**a**) without and (**b**) with the mat.

Figure 8 illustrates time history and frequency spectrum of measured results of two test conditions.

Insertion loss (IL) was used to evaluate the vibration reduction effect, defined as

(39) $$IL(f_{i})=20\mathrm{lg}\left[a_{W/O}\left(f_{i}\right)/a_{W}\left(f_{i}\right)\right]$$

where *a*_w_ and *a*_w/o_ represent the vibration responses when the slab with and without mat, respectively, and *f_i_* is the *i*-th central frequency.

**Figure 9:**
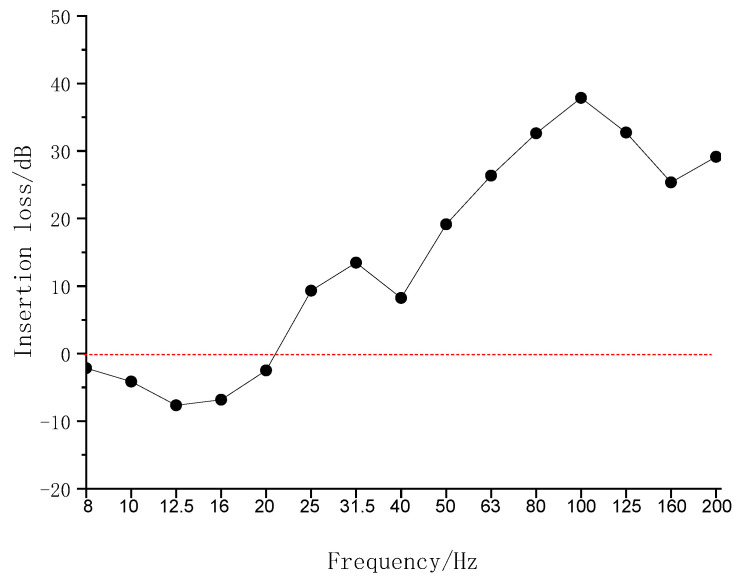
Insertion loss of tunnel wall acceleration level.

Figure 9 illustrates the test IL. It can be observed that the IL is negative below approximately 21 Hz. The natural frequency is between 12.5 and 16 Hz, at which the value of IL is the smallest, i.e., −8 dB. The largest IL is approximately 37 dB at 100 Hz.

## 4. Numerical Analysis

### 4.1. Tunnel-Soil FE Model

**Figure 10:**
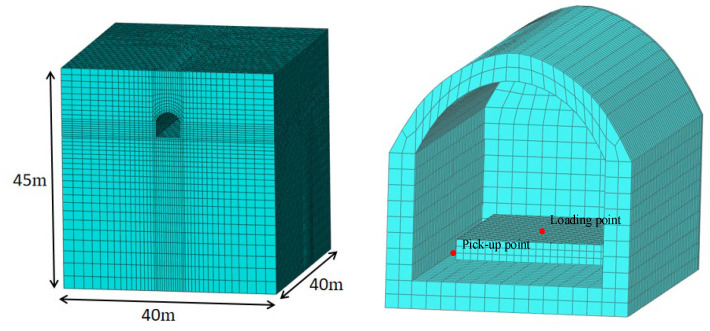
FE model of tunnel-soil.

To consider the elastic support to the underground laboratory by the soil layers, an FST-tunnel-soil FE model was built using the software ABAQUS (Figure 10). The slab and soil layers were modelled by elastic solid elements, the rails were modelled by the beam element, and the fasteners were modelled by the Kelvin’s element. According to the geological exploration result of the stratum where the underground laboratory is located, the stratum can be simplified to three layers, and the detailed parameters are listed in Table 2 To avoid the influence of the reflection of the vibration wave at the artificial truncated boundaries on the calculation results, the infinite elements CIN3D8 in ABAQUS were used around the FE model.

In the real situation, the soil is semi-infinite space. If the FE model is used to simulate infinite soil, the vibration will be reflected and scattered at an artificial boundary, which may lead to a big error of calculation. So, the infinite elements CIN3D8 can be settled in the out layer of the FE model to simulate infinite space. In ABAQUS, the infinite element CIN3D8 can be set by modifying inp. In addition, the numbering sequence should be anticlockwise and be subject to the numbering rules in ABAQUS.

The underground laboratory is a horseshoe shaped tunnel, constructed by the New Austrian Tunnelling Method. The thickness of the permanent lining is 0.55 m. The dynamic elastic modulus and mass density of the tunnel lining are 29 GPa and 2400 kg/m^3^, respectively. The embedded depth of the tunnel crown is 6 m. The whole tunnel is in the second soil layer. Both of the width and height of the internal clearance of the laboratory are 4 m.

The Rayleigh damping assumption was employed, in which the damping matrix [***C***] can be calculated by the linear superposition of mass matrix [***M***] and stiffness matrix [***K***]:

(40) $$\left[C\right]=\alpha \left[M\right]+\beta \left[K\right]$$

where *α* and *β* are coefficients, and their values are determined as 0.93396 and 2.36367 × 10^−6^.

### 4.2. Constitutive Model of the Resilient Mat

According to the actual dimension, the slab model was 7 m × 3.5 m × 0.4 m, with a dynamic elastic modulus of 31 GPa, a Possion’s ratio of 0.25, and a mass density of 2500 kg/m^3^.

**Figure 11:**
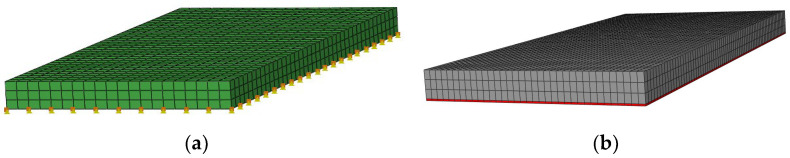
FE model of FST; the resilient mat was modelled by (**a**) Kelvin’s model and (**b**) 3PVM.

Two types of constitutive model were used to model the resilient mat: the Kelvin’s model and the 3PVM (Figure 11). In Kelvin’s model, the supporting stiffness was 0.035 N/m^3^, corresponding to the material mechanical characteristic at 10 Hz. In the 3PVM, the mat was modelled using a solid element. The coupling loss between the slab and the mat was ignored.

According to the derivation in Section 2, the 3PVM can reflect the frequency variation characteristic of the elastic modulus of the mat. The storage modulus can be expressed as:

(41) $$E=\frac{E_{1}{}^{2}E_{2}+E_{1}E_{2}{}^{2}+E_{2}\eta^{{}_{{}^{2}}}\omega^{2}}{{\left(E_{1}+E_{2}\right)}^{2}+\eta^{{}_{{}^{2}}}\omega^{2}}$$

where *ω* = 2π*f* is the angle frequency, and *E*_1_, *E*_2,_ and *η* are three parameters. According to the mechanical characteristic of the test mat, illustrated in Figure 4, when *f* = 0 Hz, *E* = 0.075 MPa; when *f* =10 Hz, *E* = 0.187 MPa; and when *f* = 30 Hz, *E* = 0.075 MPa. Then, a system of ternary nonlinear equations can be obtained:

(42) $$\left\{\begin{array}{l} \frac{E_{1}{}^{2}E_{2}+E_{1}E_{2}{}^{2}}{{\left(E_{1}+E_{2}\right)}^{2}}=0.075\\ \frac{E_{1}{}^{2}E_{2}+E_{1}E_{2}{}^{2}+E_{2}\eta^{2}{\left(20\pi \right)}^{2}}{{\left(E_{1}+E_{2}\right)}^{2}+\eta^{2}{\left(20\pi \right)}^{2}}=0.187\\ \frac{E_{1}{}^{2}E_{2}+E_{1}E_{2}{}^{2}+E_{2}{\left(60\pi \right)}^{2}}{{\left(E_{1}+E_{2}\right)}^{2}+\eta^{2}{\left(60\pi \right)}^{2}}=0.258 \end{array}\right.$$

Solving the above equations using Matlab, the value of *E*_1_, *E*_2,_ and *η* can be obtained:

(43) $$\left\{\begin{array}{l} E_{1}=0.101214\;\mathrm{MPa}\\ E_{2}=0.268431\;\mathrm{MPa}\\ \eta =0.006688\;\mathrm{MPa}\cdot s \end{array}\right.$$

Then, the elastic modulus of the mat as a function of angle frequency can be finally solved as:

(44) $$E=\frac{\begin{array}{r} 0.010042847 \end{array}+\begin{array}{r} 1.20078\times 10^{-5} \end{array}\omega^{2}}{\begin{array}{r} 0.136637375 \end{array}+\begin{array}{r} 4.47331\times 10^{-5} \end{array}\omega^{2}}$$

**Figure 12:**
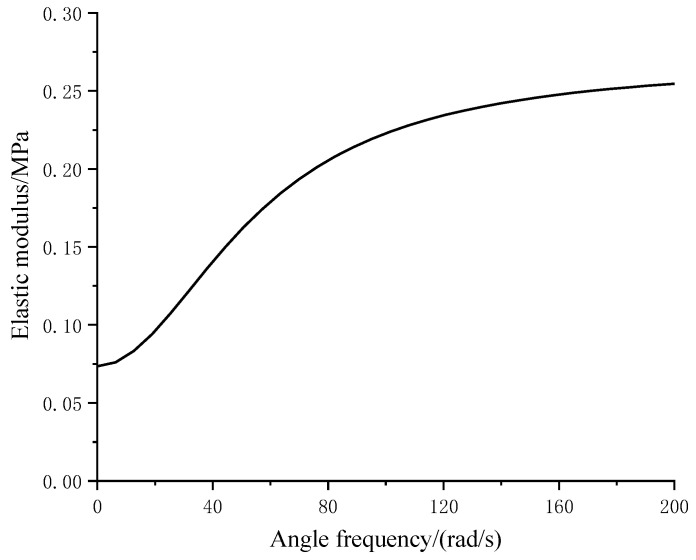
Elastic modulus varies with frequency.

Figure 12 illustrates the elastic modulus varies with angle frequency.

### 4.3. Result of Numerical Analysis

**Figure 13:**
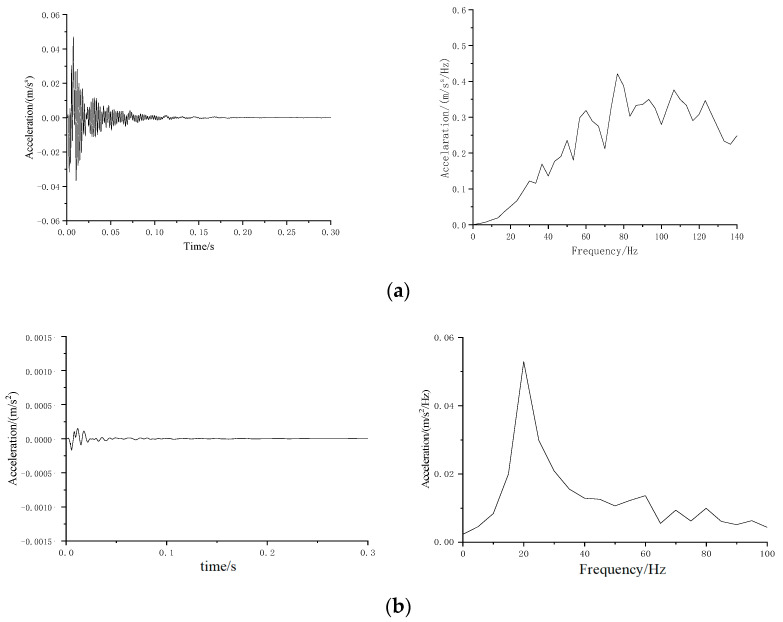

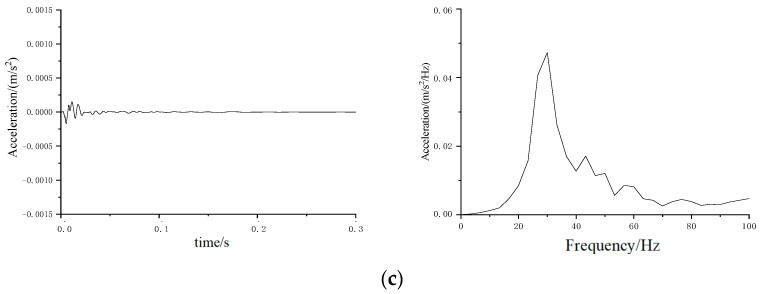
Time history and Fourier spectrum of calculated acceleration responses. (**a**) Regular slab track without mat; (**b**) FST with mat using Kelvin’s model; (**c**) FST with mat using 3PVM.

Applying the test impact force (Figure 13), the acceleration responses at the tunnel based can be calculated. Both in the time and frequency domains, the calculated responses were similar using two types of mat models.

### 4.4. Analysis of Measured and Calculated Results

**Figure 14:**
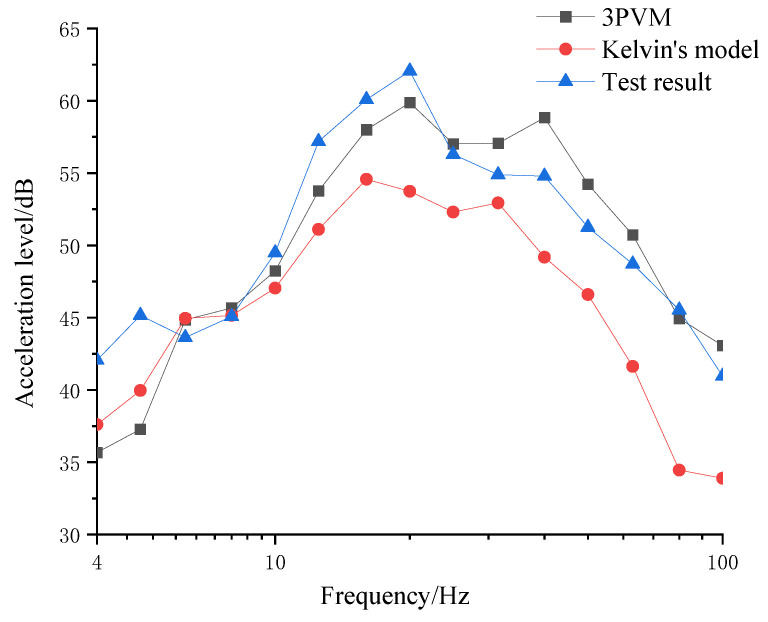
Comparison of measured and calculated 1/3 octave.

Figure 14 compared the calculated and measured vibration responses in 1/3 octave band. It can be observed that between 6 and 10 Hz, the calculated results using both models and measured results fit well. This is because both of the models can simulate the dynamic elastic modulus and dynamic stiffness around the natural frequency well. However, with the increase in frequency, the 3PVM can better reflect the relationship between the elastic modulus and frequency and the elastic modulus, and the stiffness of Kelvin’s model still maintains a fixed value, which does not fit the true mechanical property of the resilient mats. Then, the 3PVM can better reflect the true dynamic characteristics of the resilient mats than Kelvin’s model can.

Overall, Kelvin’s model result underestimates the test result at any frequency. The average error of Kelvin’s model is 5 dB, and the max error is 11 dB at 80 Hz. Compared with the test result, the 3PVM result is sometimes larger and sometimes smaller with the change of frequency. However, the average error and the max error of the 3PVM are both smaller than in Kelvin’s model.

## 5. Conclusions

To better model the resilient mat of the FST using the solid element in the FE model, a 3PVM was employed. Based on the integral constitutive equation of the 3PVM, the uniform tangent stiffness matrix of the material was derived and implemented in ABAQUS using UMAT. The calculated responses were compared with the laboratory test results. The following conclusions can be drawn:

- Overall, the 3PVM can better reflect the dynamic characteristics of the resilient mat of FST than Kelvin’s model, especially over 10 Hz.
- At 8, 25, and 80 Hz, the 3PVM can perfectly simulate laboratory test. Between 8 and 80 Hz, the values calculated by the 3PVM are smaller than the test results. Between 25 and 100 Hz, the 3PVM overestimates the true acceleration level. The average error between the 3PVM-calculated result and the test result is 2.7 dB. The max error is 7.9 dB, and it appears at 5 Hz.

In further follow-up work, more types of resilient mat materials will be simulated on the basis of this study. The research could be divided into two parts: First, for the existing material, more precise analysis could be performed to obtain clearer mastery of their characteristics. Second, some new types of materials could be developed with the aid of this modelling method. Thus, the metro-induced ground vibration could be restrained more effectively.

## Figures and Tables

**Table 1:** Detail of measuring apparatus.

Equipment	Picture	Manufacturer	Model	Main Parameters
Data acquisition equipment	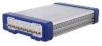	China Orient Institute of Noise and Vibration, Beijing, China	INV 3060S	Maximum sampling
Accelerometer	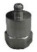	Lance Technologies Inc, Copley, OH, USA	Lance AS 0123T	Measurement range: 200 g Working frequency: 0.2 to 11,000 Hz Resolution: 0.02 mg
Accelerometer	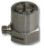	Lance Technologies Inc, Copley, OH, USA	Lance AS 0105	Measurement range:20 g Working frequency: 0.35 to 6000 Hz Resolution: 0.01 mg

**Table 2:** Soil parameters.

**Soil Type**	**Thickness/m**	**Dynamic Elastic Modulus/MPa**	**Possion’s Ratio/-**	**Mass Density/(kg/m^3^)**
**Backfill**	3	140	0.35	1650
**Sand**	30	310	0.33	2010
**Clay**	∞	670	0.29	2050

## Data Availability

The raw data, processed data and modeling codes required to reproduce these findings cannot be shared at this time, as the data also form part of an ongoing study.
